# Supplementary material for: Interrelated effects of age and parenthood on whole-brain controllability: protective effects of parenthood in mothers
Source: Front Aging Neurosci. 2023 Oct 18;15:1085153. doi: 10.3389/fnagi.2023.1085153 (PMC10618679; doi:10.3389/fnagi.2023.1085153)
Supplement: Supplementary file 1 [file Data_Sheet_1.PDF]

Supplementary Information

Interrelated effects of age and parenthood on whole-brain controllability: protective effects of parenthood in mothers

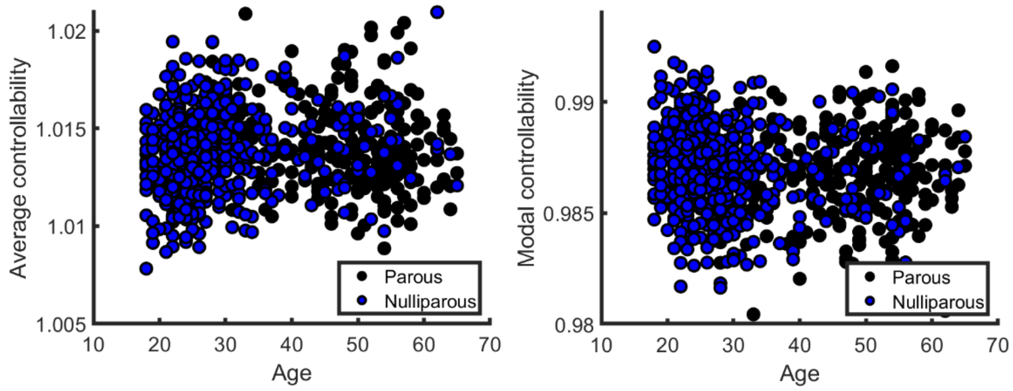

Figure S1: Distribution of average and modal controllability values for all data in this study.

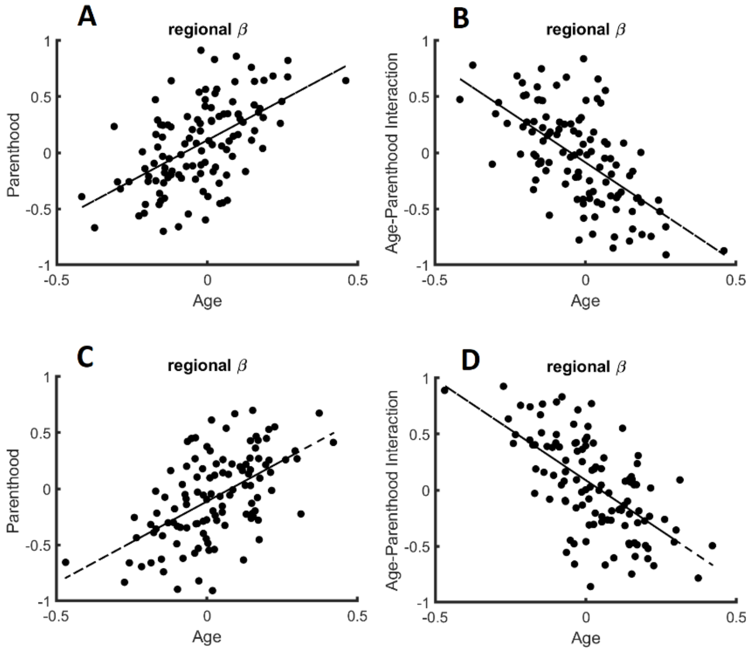

Figure S2. The relation of regional age and parenthood beta values in the linear models that predict whole-brain controllability in males. Similar to the whole-brain models of controllability, the beta values for age and parenthood have the same signs for models of average (A) and modal (C) controllability while age and age-parenthood have opposite signs in models of (B) average and (D) modal controllability.

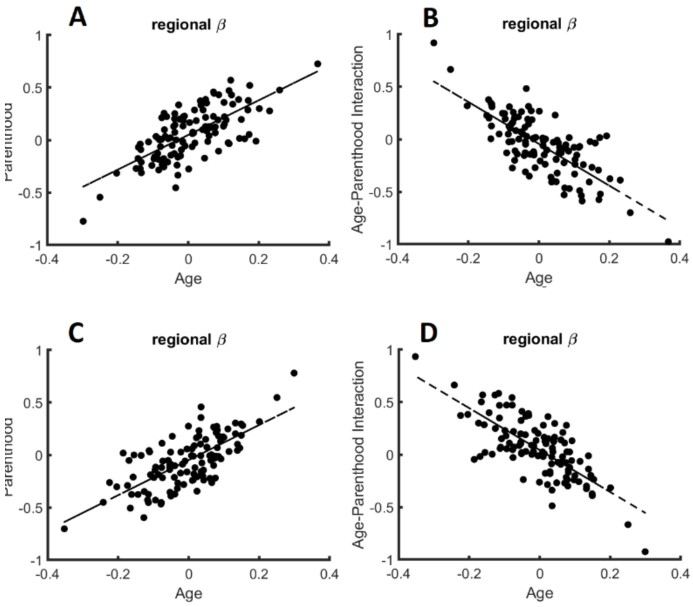

Figure S3. The relation of regional age and parenthood beta values in the linear models that predict whole-brain controllability in all subjects pooled together. Similar to the whole-brain models of controllability, the beta values for age and parenthood have the same signs for models of average (A) and modal (C) controllability while age and age-parenthood have opposite signs in models of (B) average and (D) modal controllability.

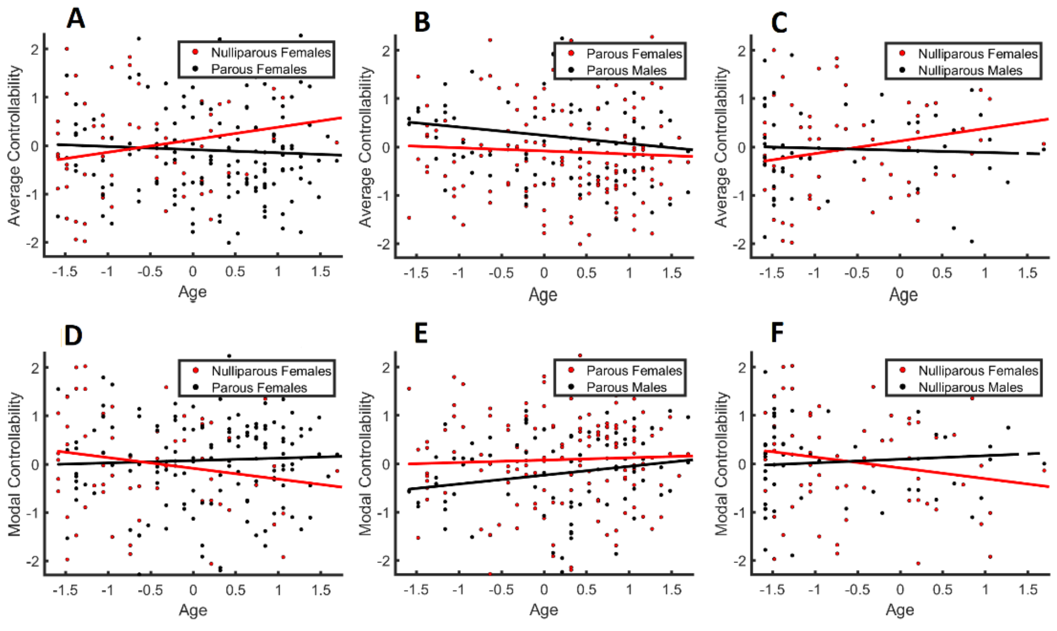

Figure S4: The relation between age, sex, and controllability together with the raw data. (A, D) Parenthood effect: average and modal controllability changes with age only in nulliparous females. (B, E) Parenthood by sex interaction: In parents, average controllability decreases with age and modal controllability increases although the effect is larger in mothers. (C, F) Parenthood by sex interaction: In non-parents, the age-related changes to controllability are more evident in nulliparous females compared to males.

Table S1: Linear model to estimate whole-brain average controllability for nulliparous women

**Model: average controllability ~ 1 + age + site + structural strength**				
	**Estimate**	**P-Value**	**T-value**	**SE**
**Intercept**	8.3e-14	1	8e-13	0.11
**Age**	0.29	0.01	2.55	0.11
**Site 1**	0.16	0.26	1.13	0.15
**Site 2**	0.21	0.15	1.45	0.15
**Structural Strength**	0.37	2e-3	3.26	0.11
umber of observations: 67, Error degrees of freedom: 62<br>Root Mean Squared Error: 0.901<br>R-squared: 0.238, Adjusted R-Squared: 0.189<br>F-statistic vs. constant model: 4.85, p-value = 0.00182				

Table S2: Linear model to estimate whole-brain modal controllability for nulliparous women

**Model: modal controllability ~ 1 + age + site + structural strength**				
	**Estimate**	**P-Value**	**T-value**	**SE**
**Intercept**	-3.2e-14	1	-2.8e-13	0.11
**Age**	-0.26	0.03	-2.21	0.12
**Site 1**	-0.14	0.33	-0.97	0.15
**Site 2**	-0.22	0.14	-1.47	0.15
**Structural Strength**	-0.35	3.7e-3	-3.01	0.12
Number of observations: 67, Error degrees of freedom: 62<br>Root Mean Squared Error: 0.918<br>R-squared: 0.209, Adjusted R-Squared: 0.158<br>F-statistic vs. constant model: 4.09, p-value = 0.00525				

Table S3: Linear model to estimate whole-brain average controllability for parous women

**Model: average controllability ~ 1 + age + site + structural strength**				
	**Estimate**	**P-Value**	**T-value**	**SE**
**Intercept**	4.2e-14	1	-1.9e-12	0.07
**Age**	-0.05	0.51	-0.66	0.07
**Site 1**	0.07	0.46	0.74	0.09
**Site 2**	0.18	0.05	2.00	0.09
**Structural Strength**	0.40	1.3e-7	5.54	0.07
Number of observations: 157, Error degrees of freedom: 152<br>Root Mean Squared Error: 0.91<br>R-squared: 0.193, Adjusted R-Squared: 0.172<br>F-statistic vs. constant model: 9.09, p-value = 1.32e-06				

Table S4: Linear model to estimate whole-brain modal controllability for parous women

**Model: average controllability ~ 1 + age + site + structural strength**				
	**Estimate**	**P-Value**	**T-value**	**SE**
**Intercept**	-3.7e-15	1	-5.06e-12	0.07

**Age**	0.04	0.62	0.49	0.07
**Site 1**	-0.08	0.4	-0.83	0.09
**Site 2**	-0.19	0.04	-2.01	0.09
**Structural Strength**	-0.39	4.2e-7	-5.29	0.07
Number of observations: 157, Error degrees of freedom: 152<br>Root Mean Squared Error: 0.917<br>R-squared: 0.18, Adjusted R-Squared: 0.158<br>F-statistic vs. constant model: 8.33, p-value = 4.21e-06				

Table S5: Full linear model to estimate whole-brain average controllability for women

**Model: average controllability ~ 1 + age + parenthood + age x parenthood + site + structural strength**				
	**Estimate**	**P-Value**	**T-value**	**SE**
**Intercept**	-2.87e-13	1	-4.76e-12	0.06
**Age**	0.31	6e-3	2.78	0.11
**Age*Parenthood**	-0.93	8e-3	-2.66	0.35
**Parenthood**	0.77	0.01	2.46	0.31
**Site 1**	0.10	0.21	1.26	0.08
**Site 2**	0.19	0.01	2.50	0.08
**Structural Strength**	0.39	6.6e-10	6.46	0.06
Number of observations: 224, Error degrees of freedom: 217<br>Root Mean Squared Error: 0.902<br>R-squared: 0.208, Adjusted R-Squared: 0.186<br>F-statistic vs. constant model: 9.49, p-value = 2.93e-09				

Table S6: Full linear model to estimate whole-brain modal controllability for women

**Model: average controllability ~ 1 + age + parenthood + age x parenthood + site +structural strength**				
	**Estimate**	**P-Value**	**T-value**	**SE**
**Intercept**	-2.58e-13	1	-4.23e-12	0.06
**Age**	-0.27	0.02	-2.40	0.11
**Age*Parenthood**	0.80	0.02	2.25	0.35
**Parenthood**	-0.66	0.04	-2.11	0.31
**Site 1**	-0.10	0.21	-1.24	0.08
**Site 2**	-0.20	0.01	-2.52	0.08
**Structural Strength**	-0.38	4.1e-9	-6.13	0.06
Number of observations: 224, Error degrees of freedom: 217<br>Root Mean Squared Error: 0.913<br>R-squared: 0.189, Adjusted R-Squared: 0.167<br>F-statistic vs. constant model: 8.45, p-value = 2.98e-08				

Table S7: Full linear model to estimate whole-brain average controllability for men

**Model: average controllability ~ 1 + age + parenthood + age x parenthood + site + structural strength**				
	**Estimate**	**P-Value**	**T-value**	**SE**
**Intercept**	2.03e-13	1	2.49e-12	0.08
**Age**	-9e-3	0.95	-0.06	0.14
**Age*Parenthood**	-0.41	0.36	-0.91	0.45
**Parenthood**	0.52	0.19	1.32	0.39
**Site 1**	0.14	0.16	1.40	0.10
**Site 2**	0.06	0.54	0.62	0.10

**Structural Strength**	0.28	1e-3	3.37	0.08
Number of observations: 138, Error degrees of freedom: 131<br>Root Mean Squared Error: 0.958<br>R-squared: 0.123, Adjusted R-Squared: 0.083<br>F-statistic vs. constant model: 3.07, p-value = 0.00769				

Table S8: Full linear model to estimate whole-brain modal controllability for men

**Model: average controllability ~ 1 + age + parenthood + age x parenthood + site + structural strength**				
	**Estimate**	**P-Value**	**T-value**	**SE**
**Intercept**	3.25e-13	1	3.97e-12	0.08
**Age**	0.04	0.77	0.29	0.14
**Age*Parenthood**	0.35	0.44	0.78	0.45
**Parenthood**	-0.48	0.22	-1.22	0.40
**Site 1**	-0.12	0.21	-1.27	0.10
**Site 2**	-0.02	0.84	-0.20	0.10
**Structural Strength**	-0.25	3e-3	-3.00	0.08
Number of observations: 138, Error degrees of freedom: 131<br>Root Mean Squared Error: 0.964<br>R-squared: 0.111, Adjusted R-Squared: 0.0703<br>F-statistic vs. constant model: 2.73, p-value = 0.0158				

Table S9: Full linear model to estimate whole-brain average controllability for women and men

**Model: average controllability ~ 1 + age + parenthood + age x parenthood + site + structural strength**				
	**Estimate**	**P-Value**	**T-value**	**SE**
**Intercept**	-2.32e-14	1	-4.79e-13	0.05
**Age**	0.17	0.04	2.02	0.09
**Age*Parenthood**	-0.70	0.01	-2.57	0.27
**Parenthood**	0.63	9e-3	2.62	0.24
**Site 1**	0.11	0.06	1.91	0.06
**Site 2**	0.14	0.02	2.37	0.06
**Structural Strength**	0.37	3.01e-13	7.58	0.05
Number of observations: 362, Error degrees of freedom: 355<br>Root Mean Squared Error: 0.921<br>R-squared: 0.166, Adjusted R-Squared: 0.152<br>F-statistic vs. constant model: 11.8, p-value = 4.7e-12				

Table S10: Full linear model to estimate whole-brain modal controllability for women and men

**Model: average controllability ~ 1 + age + parenthood + age x parenthood +site + structural strength**				
	**Estimate**	**P-Value**	**T-value**	**SE**
**Intercept**	-4.92e-13	1	-1.00e-11	0.05
**Age**	-0.14	0.12	-1.57	0.09
**Age*Parenthood**	0.59	0.03	2.14	0.28
**Parenthood**	-0.55	0.02	-2.25	0.24
**Site 1**	-0.11	0.07	-1.81	0.06
**Site 2**	-0.13	0.03	-2.14	0.06
**Structural Strength**	-0.35	6.81e-12	-7.10	0.05
Number of observations: 362, Error degrees of freedom: 355<br>Root Mean Squared Error: 0.932<br>R-squared: 0.147, Adjusted R-Squared: 0.132				

F-statistic vs. constant model: 10.2, p-value = 2.18e-10

Table S11: Full linear model to estimate whole-brain average controllability for women and men with gender as an additive covariate

**Model: average controllability ~ 1 + age + parenthood + age x parenthood +site + structural strength**				
	**Estimate**	**P-Value**	**T-value**	**SE**
**Intercept**	-2.43e-14	1	-5.03e-13	0.05
**Age**	0.18	0.04	2.05	0.09
**Age*Parenthood**	-0.71	0.01	-2.58	0.27
**Parenthood**	0.64	8e-3	2.64	0.24
**Site 1**	0.12	0.05	1.94	0.06
**Site 2**	0.14	0.02	2.40	0.06
**Structural Strength**	0.36	1.53e-12	7.33	0.05
**Gender**	-0.03	0.48	-0.71	0.05
Number of observations: 362, Error degrees of freedom: 354<br>Root Mean Squared Error: 0.922<br>R-squared: 0.167, Adjusted R-Squared: 0.151<br>F-statistic vs. constant model: 10.2, p-value = 1.35e-11				

Table S12: Full linear model to estimate whole-brain modal controllability for women and men with gender as an additive covariate

**Model: average controllability ~ 1 + age + parenthood + age x parenthood + gender + site + structural strength**				
	**Estimate**	**P-Value**	**T-value**	**SE**
**Intercept**	-4.91e-13	1	-1.0e-11	0.05
**Age**	-0.14	0.11	-1.60	0.09
**Age*Parenthood**	0.60	0.03	2.16	0.28
**Parenthood**	-0.56	0.02	-2.28	0.24
**Site 1**	-0.11	0.07	-1.84	0.06
**Site 2**	-0.13	0.03	-2.18	0.06
**Structural Strength**	-0.35	2.94e-11	-6.87	0.05
**Gender**	0.03	0.50	0.67	0.05
umber of observations: 362, Error degrees of freedom: 354<br>Root Mean Squared Error: 0.932<br>R-squared: 0.148, Adjusted R-Squared: 0.131<br>F-statistic vs. constant model: 8.77, p-value = 5.99e-10				

Table S13: Replication analysis of linear models with different age intervals. For each model, we replicated the results for data with age > age-limit where the age-limit was increased from 18 to 50 years old.

	β_{age} **with age > 30**	β_{age} **with age between [18 50] years old**
**Table S1**	0.29	0.31±0.14
**Table S2**	-0.26	-0.28±0.14
**Table S3**	-0.05	-0.06±0.03
**Table S4**	0.04	0.03±0.03

**Table S5**	0.31	0.27±0.09
**Table S6**	-0.27	-0.25±0.09
**Table S7**	-9e-3	-0.02±0.11
**Table S8**	0.04	0.03±0.10
**Table S9**	0.17	0.19±0.06
**Table S10**	-0.14	-0.17±0.07
**Table S11**	0.18	0.19±0.06
**Table S12**	-0.14	-0.17±0.07
